# Supplementary material for: Guanfacine Normalizes the Overexpression of Presynaptic α-2A Adrenoceptor Signaling and Ameliorates Neuropathic Pain in a Chronic Animal Model of Type 1 Diabetes
Source: Pharmaceutics. 2022 Oct 10;14(10):2146. doi: 10.3390/pharmaceutics14102146 (PMC9609777; doi:10.3390/pharmaceutics14102146)
Supplement: Supplementary file 1 [file pharmaceutics-14-02146-s001.zip › pharmaceutics-1806817-supplementary.pdf]

Guanfacine Normalizes the Overexpression of Presynaptic α-2A Adrenoceptor Signaling and Ameliorates Neuropathic Pain in a Chronic Animal Model of Type 1 Diabetes

Neha Munawar [1], Joelle Nader [2,†], Najat H. Khadadah [1,†], Ashraf Al Madhoun [3,4,†], Waleed Al-Ali [5], Linu A. Varghese [1], Willias Masocha [6], Fahd Al-Mulla [3] and Milad S. Bitar [1,*]

Supplementary Figures and Legends

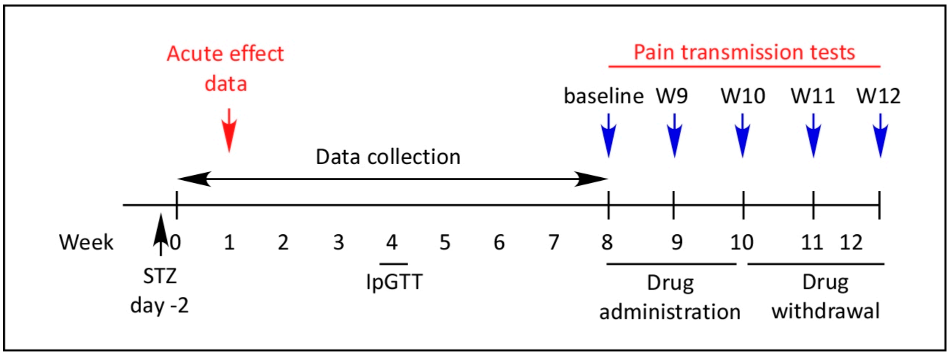

Supplementary Figure S1. Experimental procedures: the timeline of drugs administration and pain transmission tests conducted. Abbreviations: STZ, streptozotocin; and W, weeks.

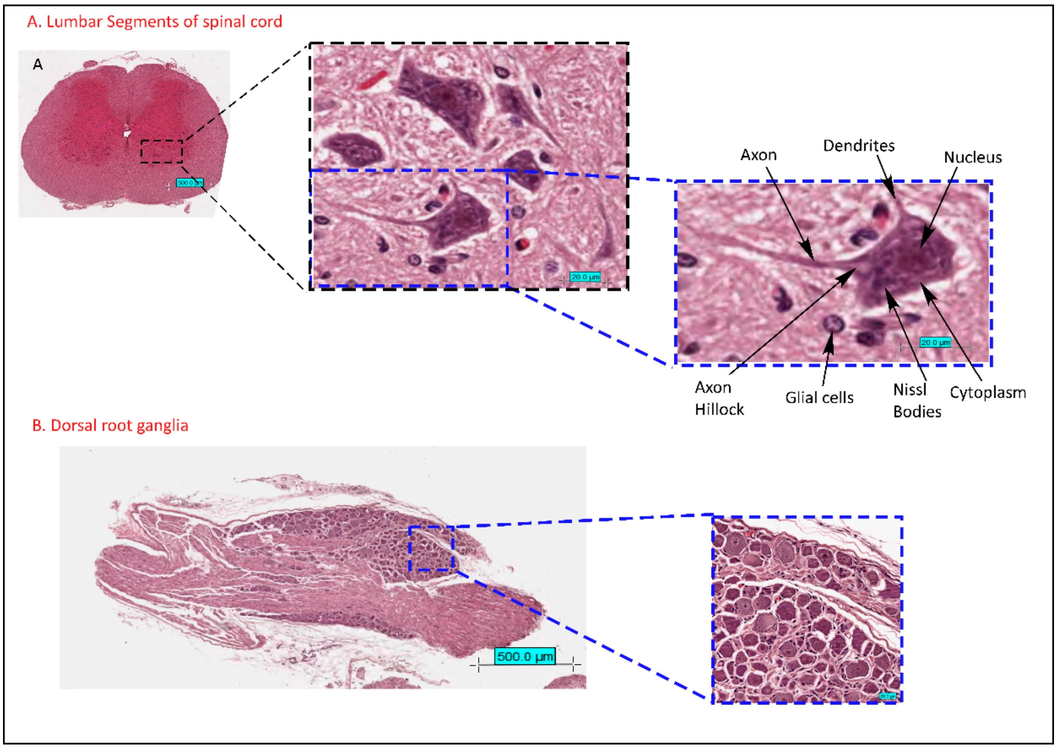

Supplementary Figure S2. Anatomy of H&E stained neuronal tissues of control rat showing the different type of cells. Lumbar segment of the spinal cord **(A)** and dorsal root ganglia **(B)**.

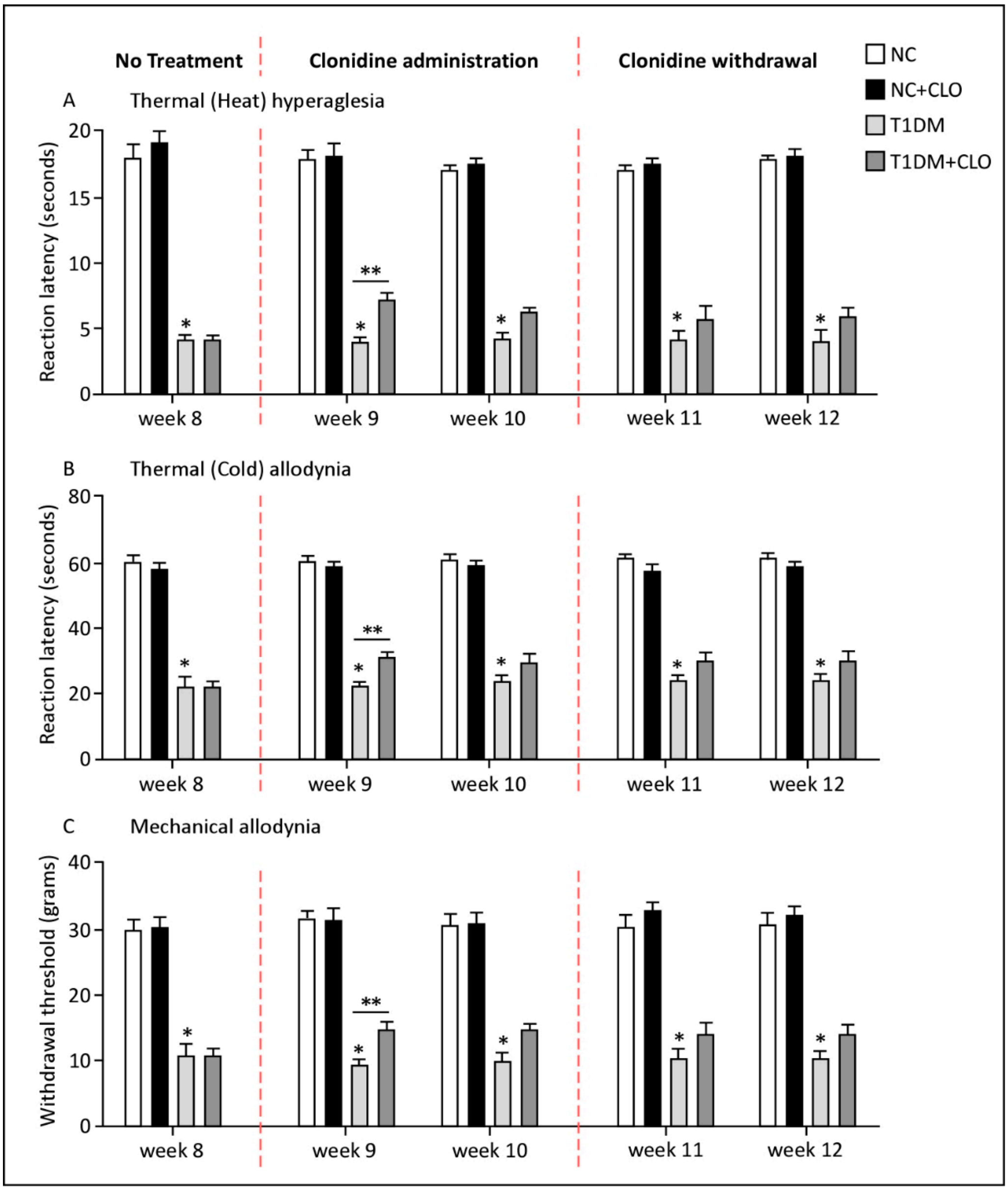

Supplementary Figure S3. Clonidine's analgesic effects following two weeks of withdrawal. Clonidine (0.1 mg/Kg) was administered intraperitoneally daily for a period of two weeks. Pain transmission tests were performed at weekly interval for two weeks of clonidine administration followed by two weeks of drug withdrawal. Data are expressed as means ± SEM obtained from five animals/group. * Significantly different from corresponding control values at $p \leq 0.05$. ** Significantly different from corresponding diabetic values at $p \leq 0.05$. Abbreviations: **NC, Normal Control; T1DM, Type 1 Diabetes Mellitus; and CLO, clonidine**.

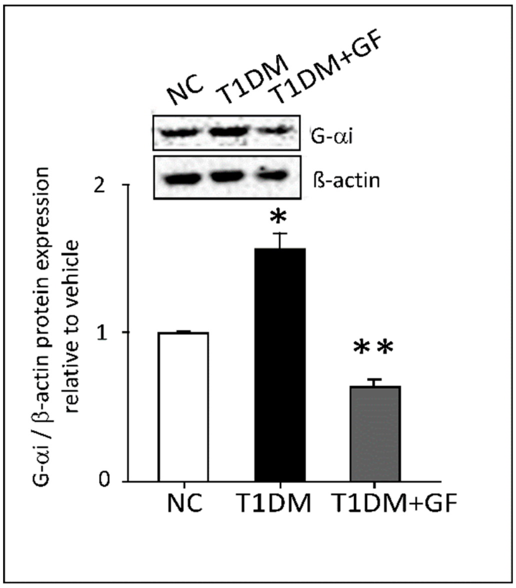

Supplementary Figure S4. Chronically administered guanfacine reversed diabetes-induced dysregulation of neuronal spinal α2-AR signaling. GPCR-subtype: G-α_i in spinal cord lumbar region. Guanfacine (0.6 mg/Kg) was administered intraperitoneally daily for a period of two weeks. Protein bands imaged with the ChemiDoc™ MP were analyzed using the Image Lab software. Data are expressed as means ± SEM from five animals/group. * Significantly different from corresponding control values at $p \leq 0.05$. ** Significantly different from corresponding diabetic values at $p \leq 0.05$. Abbreviations: **NC, Normal Control; T1DM, Type 1 Diabetes Mellitus; GF, guanfacine; G-α_i, G-protein coupled receptor G-alpha I; and α2-AR, alpha-2A adrenoceptors.**
